# *In Vitro* Surfactant Structure-Toxicity Relationships: Implications for Surfactant Use in Sexually Transmitted Infection Prophylaxis and Contraception

**DOI:** 10.1371/journal.pone.0019850

**Published:** 2011-05-16

**Authors:** Ângela S. Inácio, Katia A. Mesquita, Marta Baptista, João Ramalho-Santos, Winchil L. C. Vaz, Otília V. Vieira

**Affiliations:** 1 Center for Neuroscience and Cell Biology, University of Coimbra, Coimbra, Portugal; 2 Department of Life Sciences, University of Coimbra, Coimbra, Portugal; 3 Human Reproduction Service, University Hospitals of Coimbra, Coimbra, Portugal; 4 Department of Chemistry, University of Coimbra, Coimbra, Portugal; University of Sao Paulo, Brazil

## Abstract

**Background:**

The need for woman-controlled, cheap, safe, effective, easy-to-use and easy-to-store topical applications for prophylaxis against sexually transmitted infections (STIs) makes surfactant-containing formulations an interesting option that requires a more fundamental knowledge concerning surfactant toxicology and structure-activity relationships.

**Methodology/Principal Findings:**

We report *in vitro* effects of surfactant concentration, exposure time and structure on the viability of mammalian cell types typically encountered in the vagina, namely, fully polarized and confluent epithelial cells, confluent but non-polarized epithelial-like cells, dendritic cells, and human sperm. Representatives of the different families of commercially available surfactants – nonionic (Triton X-100 and monolaurin), zwitterionic (DDPS), anionic (SDS), and cationic (C_n_TAB (n = 10 to 16), C_12_PB, and C_12_BZK) – were examined. Triton X-100, monolaurin, DDPS and SDS were toxic to all cell types at concentrations around their critical micelle concentration (CMC) suggesting a non-selective mode of action involving cell membrane destabilization and/or destruction. All cationic surfactants were toxic at concentrations far below their CMC and showed significant differences in their toxicity toward polarized as compared with non-polarized cells. Their toxicity was also dependent on the chemical nature of the polar head group. Our results suggest an intracellular locus of action for cationic surfactants and show that their structure-activity relationships could be profitably exploited for STI prophylaxis in vaginal gel formulations. The therapeutic indices comparing polarized epithelial cell toxicity to sperm toxicity for all surfactants examined, except C_12_PB and C_12_BZK, does not justify their use as contraceptive agents. C_12_PB and C_12_BZK are shown to have a narrow therapeutic index recommending caution in their use in contraceptive formulations.

**Conclusions/Significance:**

Our results contribute to understanding the mechanisms involved in surfactant toxicity, have a predictive value with regard to their safety, and may be used to design more effective and less harmful surfactants for use in topical applications for STI prophylaxis.

## Introduction

Sexually transmitted infections (STIs) are a major public-health problem worldwide. Direct treatment costs and serious collateral perinatal damage caused by STIs represent hefty financial and social burdens, particularly in developing countries [1]. The World Health Organization estimates 340 million new cases of bacterial (gonorrheal, syphilitic, and chlamydial infection) and protozoan (trichomoniasis) STIs per year [2], a number that does not include the millions of new STIs with with fungal (candidiasis) and viral etiology (herpes simplex type 2 (HSV-2), hepatitis B (HBV), papilloma (HPVs) and immunodeficiency (HIV)). In recent years, HIV infections in women constitute more than half of the new infections, a result of their greater biological [3] and social vulnerability [4]. Population growth linked to unintended pregnancies also represents a serious social problem and a hindrance to improvement of living conditions in less developed regions of the world. The correct and persistent use of condoms provides a high level of protection against all STIs and unplanned pregnancies, but many women lack the social and/or economic power to persuade their partners to use them. Consequently, there is an urgent need for new woman-controlled prevention methods. Topical applications (such as vaginal gels) with microbicidal and spermicidal activity, that can be used by women without the need for consent of a male partner [5] are one possible answer to the problem. Ideally these topical applications should be highly effective, non-disruptive of the integrity of the vaginal epithelial barrier, should not induce mucosal inflammation, not interfere with the innate immune response nor alter vaginal flora, and, particularly for the poorer regions of the world, be cheap, stable, easy to store and easy to use.

The bacteriostatic and bactericidal actions of surfactants have been recognized for many years. Surfactant-based devices for purposes of contraception have been in use since decades and the first microbicidal vaginal gels to be tested in clinical trials were surfactant-based. However, all the surfactant-based microbicide candidates that completed phase III clinical trials failed to prevent HIV infection and their utility as general microbicides was also questionable. The first to fail was nonoxynol-9 (N-9), a non-ionic surfactant widely used as spermicide, that in pre-clinical studies seemed to provide protection against some STIs [6], [7] and destroyed HIV *in vitro* [8] but in clinical studies induced irritation of the vaginal mucosa and facilitated HIV transmition [9], [10]. The failure of N-9 was followed by C31G, a mixture of a zwitterionic and a nonionic surfactant, in a formulation known as “SAVVY Vaginal Gel” [11], [12]. More recent phase III studies, with nonspecific microbicide gels that did not include surfactants (Carraguard and PRO2000) also did not demonstrate efficacy. These disappointing results pointed to the urgency in understanding the detailed biological mechanisms responsible for microbicide toxicity and the necessity to develop new *in vitro* models and safety biomarkers, in order to improve the prediction of clinical outcomes in large-scale efficacy trials in the future.

The initial discouraging results of microbicide clinical trials turned the main focus of current research to the development of formulations containing specific antiretroviral drugs [13]. However, it cannot be ignored that HIV is a highly mutable virus and is known to develop resistance to antiretroviral drugs [14], [15], [16]. Although at present, and from the perspective of richer societies, prophylaxis against HIV infection steals the limelight, the targeting of STIs with bacterial and protozoan etiologies are of major importance because the inflammation caused by these infections not only has a direct impact on human health but also facilitates and increases HIV transmission [11], [12].

*In vitro* studies reported previously from our laboratory showed that cationic surfactants may work as bactericides at concentrations that are not harmful to polarized mammalian epithelial cells [17]. Thus, despite the negative results of the clinical trials referred above, a step-by-step, systematic investigation of the toxicity of different types of surfactants towards mammalian cells (particularly polarized epithelial cells and other cell types encountered in the vaginal mucosa) seems to be warranted. It is particularly important to build up a dependable data base which enables an understanding of the structure-toxicity relationships and the mechanisms of surfactant toxicity with regard to the mammalian cells and the microbial infecting agents. Such a data base can be expected to be helpful in designing new, more effective, and less toxic amphiphile molecules with potential use as broad-spectrum microbicidal agents. With this objective in mind we report here the effects of concentration, exposure time and surfactant structure on the *in vitro* viability of mammalian cells that model the most vulnerable cell types that exist in the human cervicovaginal mucosa, namely, fully polarized columnar epithelial cells (MDCK and Caco-2), human cervical non-polarized epithelial cells (HeLa) and dendritic cells (FSDC). To evaluate the utility of surfactants as contraceptive agents we have also studied surfactant toxicity towards human sperm. Representatives of all families of commercially available surfactants were tested: Nonionic – Triton X-100 (TX-100) and *rac*-1-lauroylglycerol (Monolaurin); zwitterionic – N-dodecyl-N,N-dimethylammonium-propanesulfonate (DDPS); anionic – sodium dodecyl sulfate (SDS); and cationic – a homologous series of *n*-Alkyl-N,N,N-trimethylammonium bromides (C_n_TAB with n from 10 through 16), N-dodecylpyridinium bromide (C_12_PB), and Dodecyl-N-benzyl-N,N-dimethylammonium (better known as Benzalkonium) bromide (C_12_BZK). All these surfactants are commercially available and were chosen because their action as microbicides is well documented in the literature. For reasons previously discussed by us in detail [17] the surfactant effects were compared taking into account their respective Critical Micelle Concentrations (CMC).

Our results show that the toxicity of all tested surfactant families was significantly higher in non-polarized cells compared to polarized cells and the toxic effect showed a dependency on the nature of the polar head groups, cationic surfactants being the most toxic. Whereas the nonionic, zwitterionic and anionic surfactants showed toxicity at concentrations around their CMC, clearly suggesting that their toxicity is related to destabilization and/or destruction of cell membranes, cationic surfactants were toxic at concentrations well below their CMC, suggesting that their action does not necessarily involve cell membrane disruption. Cationic amphiphiles of the C_n_TAB family, differing in the length of their hydrophobic chain, exhibit a non-linear dependence of their toxicity on the hydrocarbon chain length. Among the cationic surfactants the nature of the polar head groups was also shown to be a determinant in their toxicity towards the cells examined. When the hydrocarbon chain lengths were comparable, the CMC-normalized relative toxicity scale was found to be C_12_BZK ≈ C_12_PB >> C_12_TAB. We shall discuss possible reasons for this effect. A comparison of the surfactant toxicity towards human sperm compared with the other cells tested showed that, in general, the surfactants studied in this works have very low therapeutic indices. Although C_12_PB and C_12_BZK have better therapeutic indices than all other surfactants examined, even these can, at best, be classified as having a “narrow therapeutic index”. Their use in contraceptive formulations must, therefore, be treated with caution.

## Results

### Surfactant Critical Micelle Concentrations

The formation of surfactant micelles in aqueous media may be considered, under certain conditions, to be similar to a phase separation between an aqueous solution of surfactant monomers and a micellar phase. The surfactant concentration at which this phase separation occurs is the Critical Micelle Concentration (CMC). Within a given homologous series of surfactants the CMC is linearly proportional to the free energy of partitioning of the apolar part of the surfactant between an apolar environment such as a micelle or the lipid bilayer of a cell membrane and the aqueous phase [18]. Thus, it is important to use the CMC of the surfactants as a reference concentration when comparing the toxic effects of any surfactant homologous series. In the present study the stock solutions of surfactants used to treat the cells were prepared as fractions (or multiples) of their CMC in OptiMEM serum-free cell culture media. Since the CMC can be dependent on the ionic strength and pH, the CMC of each surfactant was measured under our experimental conditions and the results are listed in Table 1.

**Table 1:** Critical Micelle Concentrations (CMC) of the surfactants used, calculated in a saline buffer (1.8 mM CaCl_2_, 0.8 mM MgSO_4_, 5.3 mM KCl, 26.2 mM NaHCO_3_, 117.2 mM NaCl and 1.0 mM NaH_2_PO_4_.H_2_O, pH 7.3).

Surfactant		CMC (M)		
		Experimental	Literature	Ref.
Non-ionic	TX-100	(2.0±0.1)×10^−4^	2.0×10^−4^	[57]
	Monolaurin	(4.2±0.5)×10^−5^	4.4×10^−5^	[62]
Zwiterionic	DDPS	(2.0±0.1)×10^−3^	2.0×10^−3^	[58]
Anionic	SDS	(2.6±0.3)×10^−4^	2.6×10^−3^	[58]
Cationic	C_10_TAB	(4.0±0.1)×10^−2^	4.0×10^−2^	[58]
	C_12_TAB	(3.5±0.3)×10^−3^	3.5×10^−3^	[58]
	C_14_TAB	(2.9±0.1)×10^−4^	2.8×10^−4^	[58]
	C_16_TAB	(2.6±0.2)×10^−5^	2.6×10^−5^	[58]
	C_12_PB	(3.9±0.1)×10^−3^	5.0×10^−3^	[63]
	C_12_BZK	(1.7±0.9)×10^−3^	5.0×10^−3^	[64]

Data are shown as mean ± SD of at least 3 independent experiments.

Note: The literature values cited are not for the saline buffer in which we obtained our results but for an aqueous phase ionic strength most closely resembling that of our buffer system.

### Effects of the surfactant type, concentration and exposure time on the viability of polarized mammalian columnar epithelial cells

The primary target for bacterial and viral sexually transmitted infections, in women, is the non-keratinized squamous epithelium of the vagina and ectocervix, as well as the single-layer columnar epithelium of the endocervix. It has been shown that the vaginal columnar epithelium is the primary site of damage in the use of surfactants [10], [19], [20]. We have, therefore, tested the effect of four classes of commercially available surfactants towards the viability of MDCK and Caco-2 columnar epithelial cell lines. These two cell lines, although not of vaginal origin, are derived from mammalian columnar epithelia and can be grown to a completely confluent and polarized state, with relatively non-leaky tight junctions, closely resembling the characteristics of the vaginal columnar epithelium. There is a vast literature on the nature and properties of these polarized epithelial cell lines in culture and they have being widely used in similar conditions as we report here [17], [21], [22], [23].

**Figure 1:**
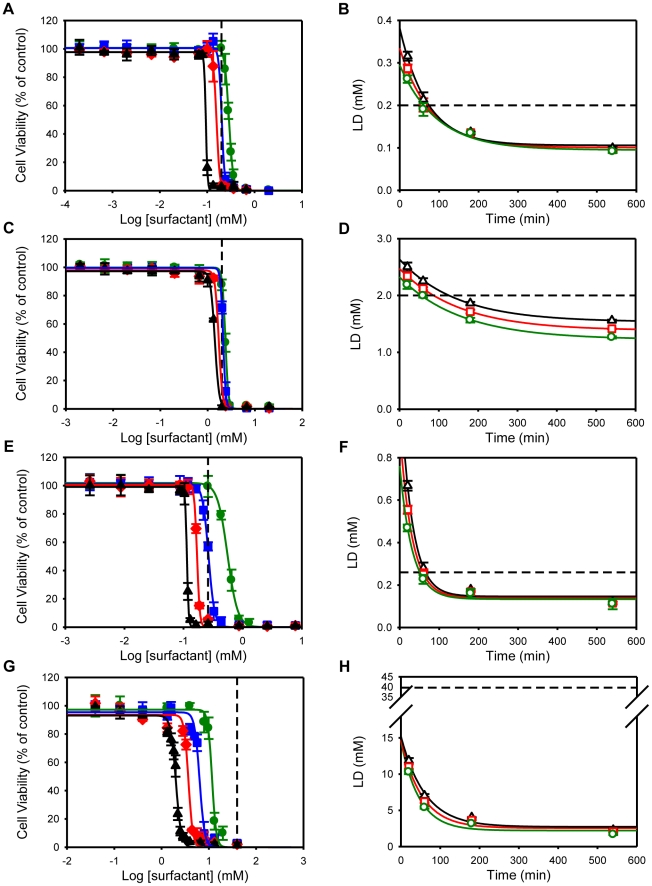
Effect of different surfactant classes on polarized and confluent MDCK cells viability. Four surfactant classes were evaluated: nonionic TX-100 (A, B), zwitterionic DDPS (C, D), anionic SDS (E, F) and cationic C_10_TAB (G, H). Cell Viability was assessed by the MTT assay 24h after the cells had been exposed to different concentrations of surfactants for 20 (green closed circles), 60 (blue closed squares), 180 (red closed diamonds) and 540 (black closed triangles) minutes (left panels). Cell viability is expressed as percentage of the viability of control cells. The data of each independent experiment was fitted to a four parameter logistic equation and the LD_10_ (green open circles), LD_50_ (red open squares) and LD_90_ (black open triangles) concentrations determined for each time point (right panels). A mono-exponential decay equation was fitted to the LD *vs.* exposure-time curves and for each surfactant tested the decay constants calculated for the LD_90_, LD_50_ and LD_10_ curves were similar. The CMC of each surfactant is represented by the black dashed line. Data are presented as mean ± SD of at least 3 independent experiments, each one done in triplicate.

MDCK and Caco-2 cells were exposed to different concentrations of surfactants during 20, 60, 180 and 540 minutes. The surfactants studied were: the nonionic TX-100 and Monolaurin, the zwitterionic DDPS, the anionic SDS and the cationic C_10_TAB. Cell toxicity was measured by the MTT assay 24 hours post-exposure to surfactants. The MTT assay is one of the most used cytotoxicity assays and is based on the reduction of MTT to a formazan by mitochondrial and/or cytoplasmic dehydrogenases. Cell viability is expressed as percentage of the viability of mock-treated control cells. The data of each independent experiment was fitted to a four parameter logistic equation [24], [25] and the Lethal Dose 90 (LD_90_), Lethal Dose 50 (LD_50_) and Lethal Dose 10 (LD_10_), surfactant concentrations at which cell viability was, respectively, 10%, 50% and 90% of the control, were determined for each exposure time (we have determined the LD_10_, LD_50_ and LD_90_ values for all the surfactants and all cells types reported on in this paper; interested readers are invited to contact us directly for this data). The LD *vs.* exposure-time curves for each surfactant tested could be fitted to a mono-exponential decay equation and the decay constants recovered for the LD_90_, LD_50_ and LD_10_ curves were similar (Table 2), which indicates that the mechanism(s) that cause(s) the death of 10% of the cells is the same as that responsible for killing 50 and 90% of the cells. All surfactants used in this study revealed concentration and time-dependent toxic effects. The results also showed that surfactants exhibited different degrees of toxicity depending on the nature of the polar head. For TX-100, DDPS and SDS, cytotoxicity was not observed up to concentrations close to the CMC (Figure 1 A–F), whereas the toxicity of C_10_TAB was at concentrations that were much lower than its CMC (Figure 1 G and H), the LD_50_ for both, MDCK and Caco-2 cells, being 0.05 × CMC after 540 minutes exposure. However, despite the higher toxicity of C_10_TAB when compared to the other surfactants tested, previous results from our laboratory showed that cationic surfactants are even more toxic to bacterial infectious agents than they are to columnar epithelial cells [17] and thus should be considered for development as bactericidal agents in the prophylaxis of STI that have a bacterial etiology. On the contrary, despite their low toxicity, TX-100, DDPS and SDS were shown to have neither bactericidal nor spermicidal activity at concentrations that were not harmful to epithelial cells [17]. We have also tested the toxicity of the nonionic surfactant monolaurin since there are several studies that demonstrated its bactericidal efficacy [26], [27], [28]. Up to a concentration of 10 × CMC monolaurin was neither toxic to MDCK nor to Caco-2 cells (data not shown), which may justify more detailed studies of this surfactant in the future.

**Table 2:** Decay constants (min^−1^) calculated for the exposure-time-dependence of the LD_90_, LD_50_ and LD_10_ concentrations.

Surfactant			Decay Constant (min^−1^)			
			MDCK	Caco-2	HeLa	FSDC
Non-ionic	TX-100	LD_90_	(1.28±0.10)×10^−2^	(4.33±0.80)×10^−2^	(2.07±0.76)×10^−2^	(10.0±1.00)×10^−3^
		LD_50_	(1.13±0.15)×10^−2^	(3.87±0.78)×10^−2^	(2.07±0.67) X 10^−2^	(8.67±0.58)×10^−3^
		LD_10_	(1.03±0.15)×10^−2^	(3.70±1.01)×10^−2^	(2.00±1.01)×10^−2^	(8.00 ± 1.00)×10^−3^
Zwiterionic	DDPS	LD_90_	(7.33±0.58)×10^−3^	(7.18±5.88)×10^−3^	(8.33±2.52)×10^−3^	(7.00±2.83)×10^−3^
		LD_50_	(7.00±1.00)×10^−3^	(6.91±3.59)×10^−3^	(9.00±4.36)×10^−3^	(5.06±3.35)×10^−3^
		LD_10_	(7.00±1.00)×10^−3^	(7.99±1.88)×10^−3^	(9.33±4.93)×10^−3^	(5.00±1.00)×10^−3^
Anionic	SDS	LD_90_	(3.40±0.62)×10^−2^	(1.57±0.15)×10^−2^	(1.87±0.32)×10^−2^	(8.00±2.00)×10^−3^
		LD_50_	(3.28±0.69)×10^−2^	(2.03±0.40)×10^−2^	(1.73±0.59)×10^−2^	(8.67±3.79)×10^−3^
		LD_10_	(3.22±0.79) ×10^−2^	(2.50±0.46) ×10^−2^	(1.77±0.93) ×10^−2^	(9.33±2.31) ×10^−3^
Cationic	C_10_TAB	LD_90_	(1.70±0.30) ×10^−2^	(2.10±0.89) ×10^−2^	(9.33±0.58) ×10^−3^	(1.20±0.27) ×10^−2^
		LD_50_	(1.97±0.25) ×10^−2^	(2.40±0.36) ×10^−2^	(8.67±1.53) ×10^−3^	(1.53±0.32)×10^−2^
		LD_10_	(2.23±0.31)×10^−2^	(2.60±0.44)×10^−2^	(7.67±1.15)×10^−3^	(1.90±0.79)×10^−2^

Data are shown as mean ± SD of at least 3 independent experiments.

### Surfactant toxicity towards human epithelial-like HeLa cells

**Figure 2:**
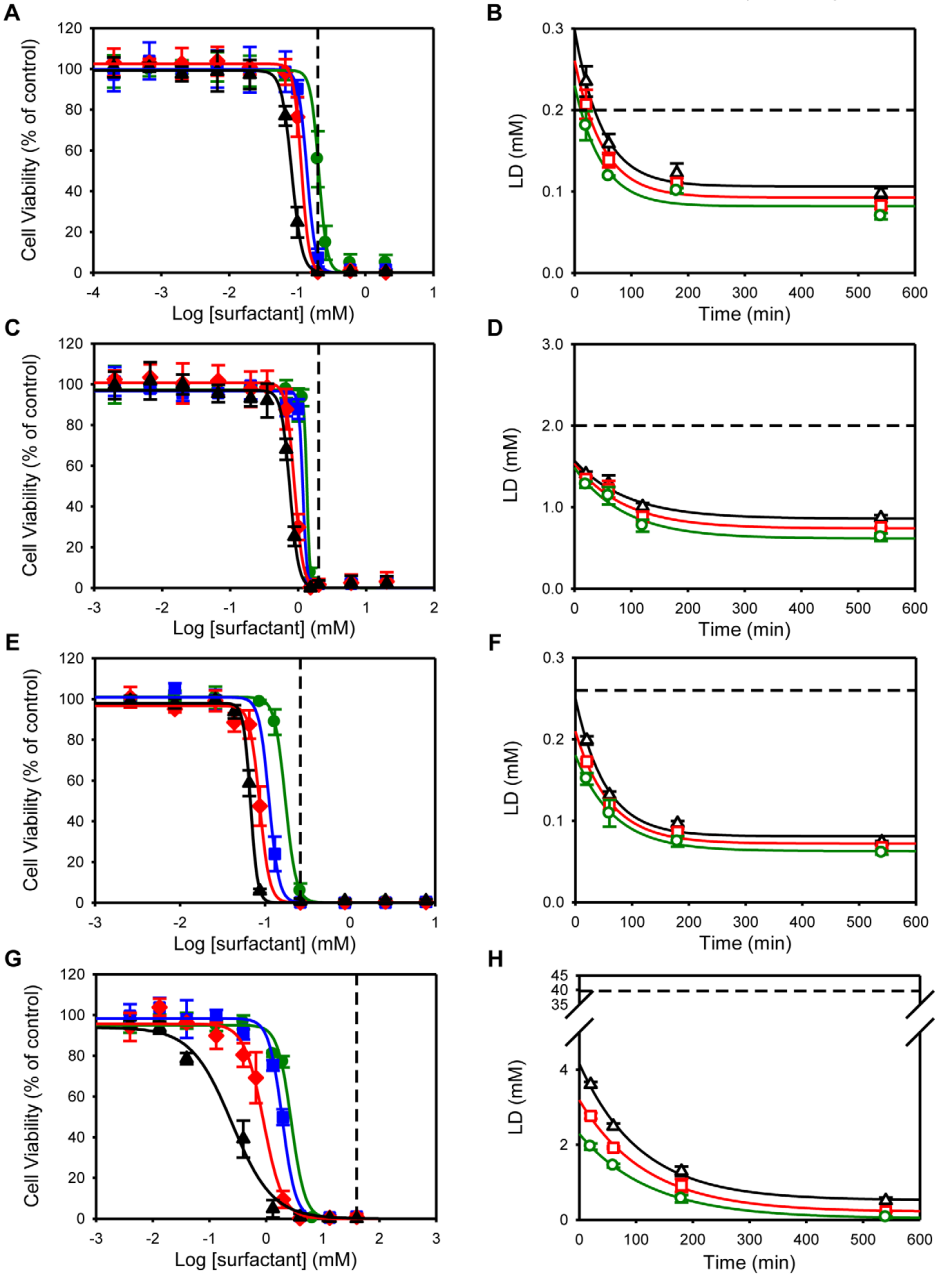
Effect of different surfactant classes on confluent HeLa cells viability. All data is as detailed in Figure 1.

The toxic effects of surfactants were also tested in confluent HeLa cell cultures as described for polarized epithelial cells. This cell line was chosen since it is a human cervical cell line. Despite the fact that these cells have epithelial origin, they are usually referred to as epithelial-like cells because they do not completely polarize and do not establish tight junctions between them. As observed with polarized columnar epithelial cells, the decay constants calculated for the exposure-time dependence of LD_90_, LD_50_ and LD_10_ were similar for all the surfactants tested (Table 2). Moreover, the results show a similar trend as described for epithelial polarized cells, in which surfactant toxicity was dependent on the nature of the polar head. As in the case of polarized epithelial cells TX-100, DDPS and SDS induced cell toxicity at concentrations close to the CMC (Figure 2). Although for short exposure times (20 and 60 minutes) these surfactants were more toxic to HeLa than to polarized epithelial cells, at longer exposure times the toxic concentrations are similar for all cell types. The effect of the nonionic monolaurin was once more tested and, as described above for polarized epithelial cells, it did not induce cell toxicity at concentrations up to 10 times CMC (data not shown). In the case of the cationic C_10_TAB the difference in cell toxicity between polarized and non-polarized cells, as seen by the LD_50_, was quite pronounced and persisted for longer exposure times (Figure 1 and 2). The LD_50_ of C_10_TAB after 540 minutes of incubation was 0.006 × CMC, which is 10 times less than for MDCK and Caco-2 cells. This difference may be due to one or both of the following facts: 1) The apical membrane of polarized cells is more ordered than the non-polarized membrane of HeLa cells; 2) The total plasma membrane surface of polarized epithelial cells is 8 times higher than that of non-polarized cells due to the extensive microvillation of the more ordered apical membrane domain in epithelial cells [29]. Surfactant toxicity is dependent upon the ability to partition between the aqueous phase and the cell membrane and may also depend upon their ability to subsequently cross the membrane and enter the cytoplasm. The fact that amphiphile partition coefficients as well as the rate constants for their insertion into membranes and translocation across them are lower for more ordered membranes [30], [31], [32], [33] explains why HeLa cells, that are neither polarized nor have well-formed tight junctions to make their less ordered membrane domains less accessible, may be more susceptible to surfactant toxicity than fully polarized and confluent epithelial cells such as MDCK and Caco-2 cell lines.

### Effects of the surfactants on the viability of mammalian dendritic cells

**Figure 3:**
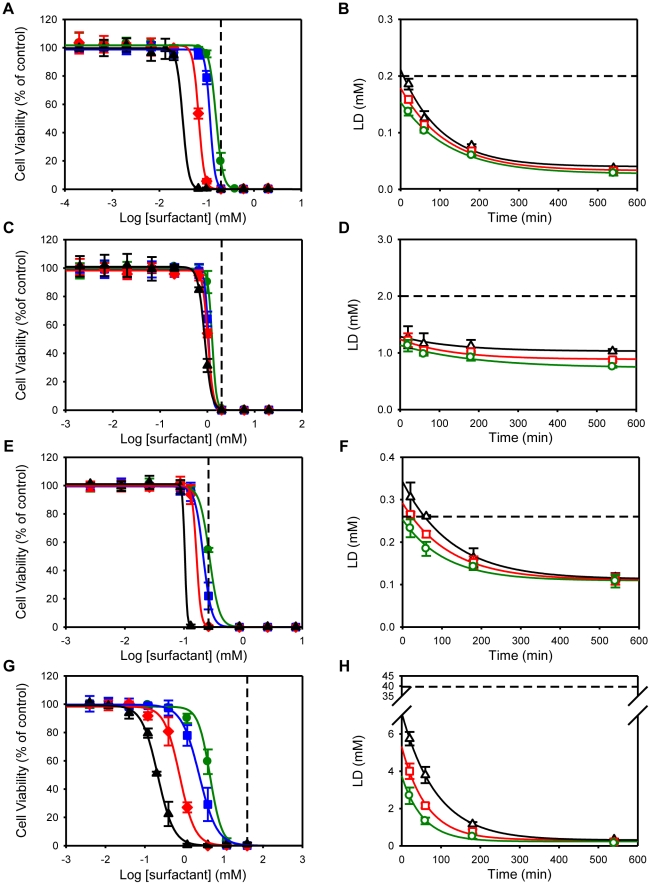
Effect of different surfactant classes on FSDC dendritic cells viability. All data is as detailed in Figure 1.

The human cervicovaginal mucosa contains the full spectrum of cell types and immune modulators that comprise both, the innate and adaptive immune system, that are necessary for an effective response against viral and bacterial infections. Among these, dendritic cells are of particular interest because of their ability to sense and process pathogens and to present viral antigens, including HIV, to T cells [34], [35], [36]. An ideal microbicide should not induce mucosal inflammation nor interfere with the innate immune responses. Because of that, the toxic effect of surfactants towards a dendritic cell line (FSDC) was tested. FSDC cells were treated in the same way as described above for MDCK, Caco-2 and HeLa cells, but at the time of the exposure to the surfactants the cells were only 70–80% confluent. The results obtained (Figure 3) were similar to the results for HeLa cells. However, FSDC were more sensitive to the effect of the nonionic TX-100 and monolaurin surfactants. In these cells a monolaurin concentration of 10 × CMC reduced cell viability by almost 30% after 540 minutes exposure (data not shown).

Rescigno et al. [37] have demonstrated that dendritic cells can open the tight junctions between epithelial cells, send dendrites outside the epithelium and directly sample bacteria without leaving the *lamina propria*. Being so, it is possible that *in vivo* these cells are less exposed to the surfactants than in our *in vitro* model and the *in vivo* toxic effects may be lower than reported here.

### Effects of cationic surfactant structure upon the viability of different types of mammalian cells

Despite the fact that clinical trials using surfactant-based gels have failed, it has been shown that several quaternary ammonium compounds, with various alkyl chain lengths and polar head groups, exert antibacterial activity against both Gram positive and Gram negative bacteria, as well as against some pathogenic species of fungi and protozoa, at concentration that are not harmful to mammalian epithelial cells [17], [38]. The study of the relation between cationic surfactant structure and its toxic effects is crucial to understand the mechanisms involved in surfactant toxicity and make predictions of the impact that new surfactants will have in cell viability. For this reason we tested the effects of the hydrocarbon chain length and polar head group structure of the cationic surfactants upon cell viability. To do so, we treated the cells with surfactants of a homologous series of cationic Alkyl-N,N,N-trimethylammonium bromides (C_10–16_TAB), C_12_PB and C_12_BZK.

**Figure 4:**
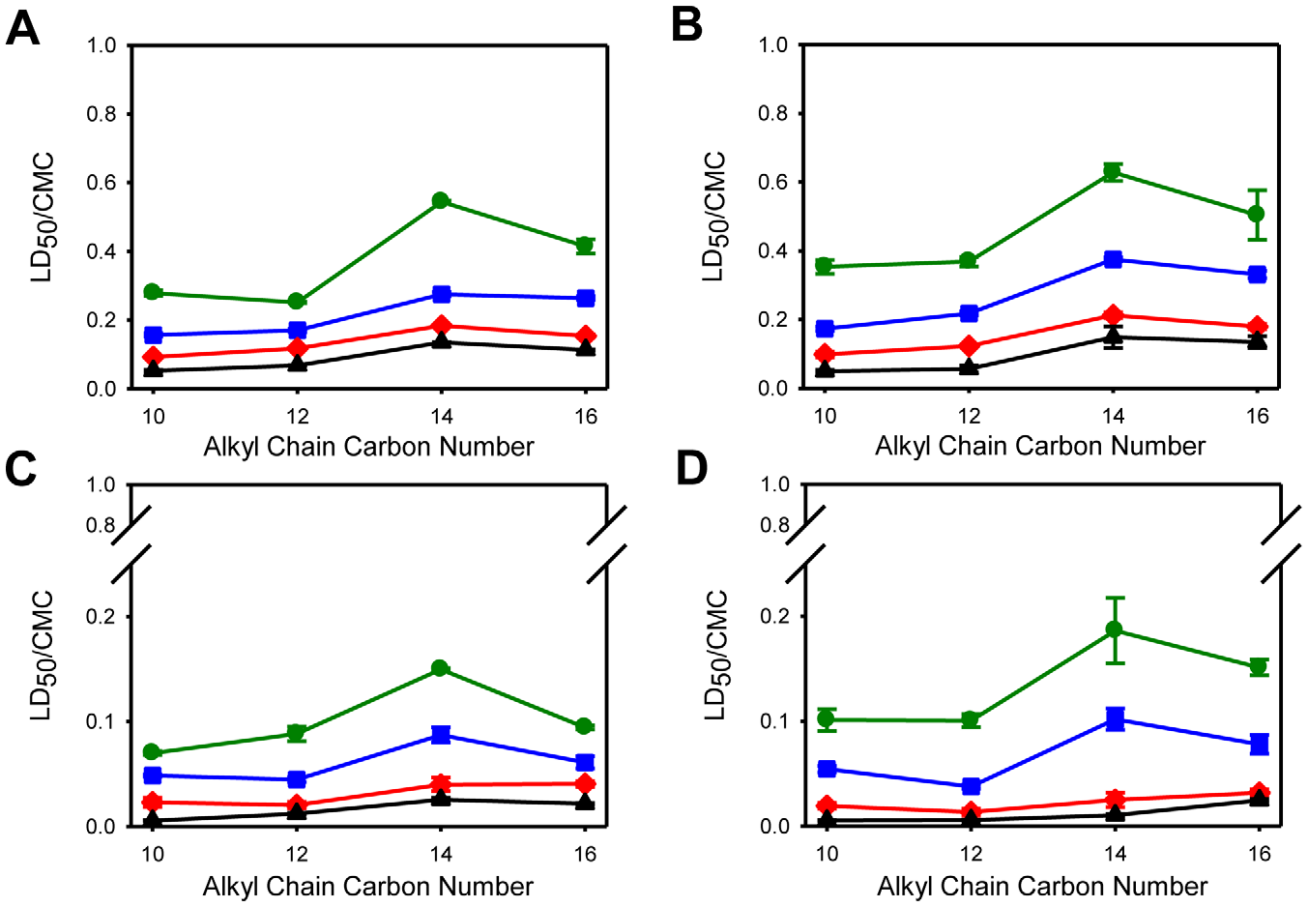
Effect of C_n_TAB surfactants hydrophobic chain length upon cell viability. The graphs show the LD_50_ concentration of the surfactants C_10–16_TAB for the polarized epithelial cells, MDCK (A) and Caco-2 (B), epithelial-like HeLa cells (C) and FSDC dendritic cells (D) after exposure times of 20 (green closed circles), 60 (blue closed squares), 180 (red closed diamonds) and 540 (black closed triangles) minutes. LD_50_ concentrations of each surfactant are normalized with respect to the CMC. Data are presented as mean ± SD of at least 3 independent experiments, each of which was done in triplicate.

**Figure 5:**
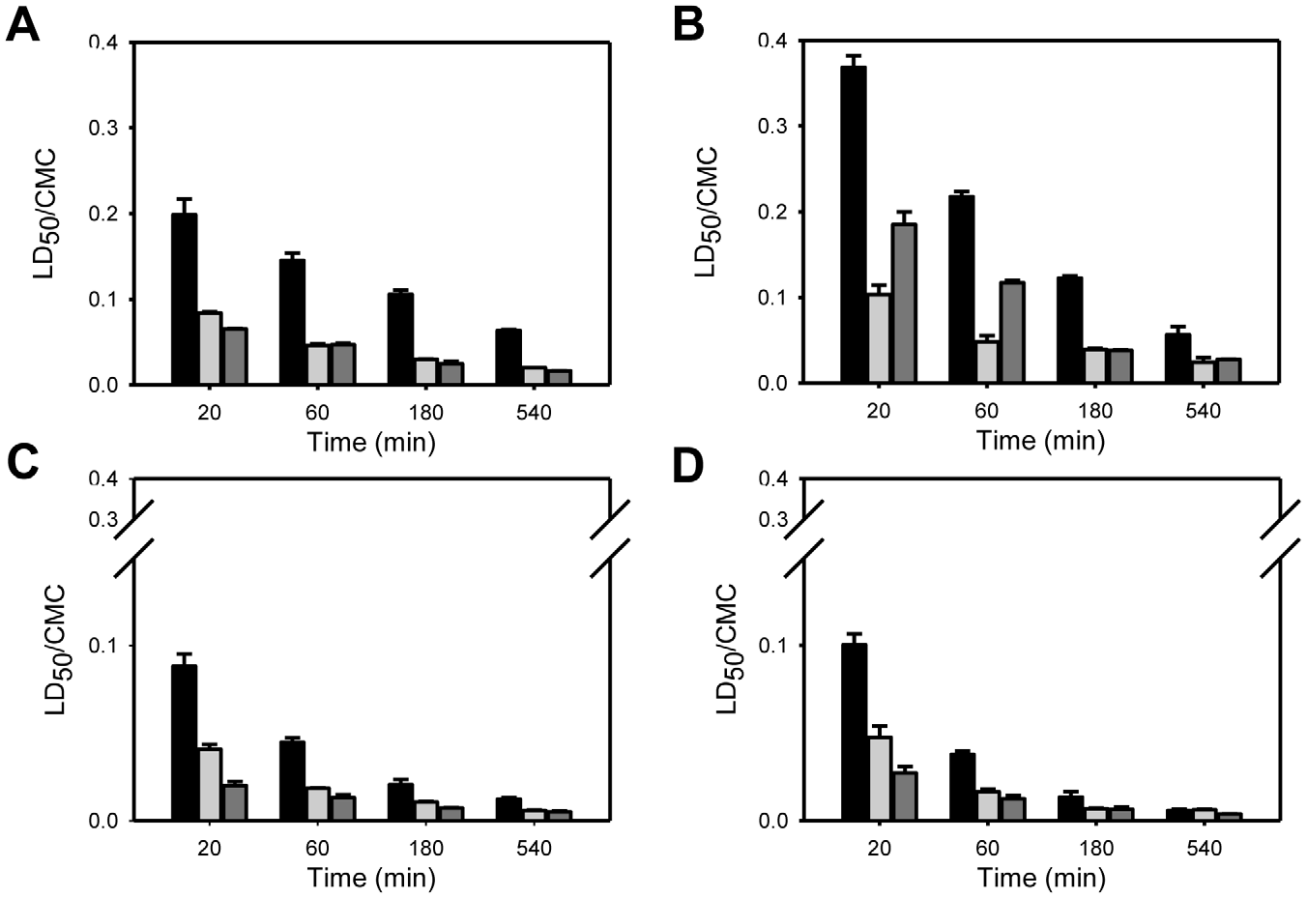
Effect of the polar head structure of cationic surfactants in cell viability. The LD_50_ concentration of the three cationic surfactants with similar hydrophobic chain length, but different polar head groups, was evaluated in polarized epithelial cells, MDCK (A) and Caco-2 (B), epithelial-like HeLa cells (C) and FSDC dendritic cells (D). The surfactants tested were C_12_TAB (black bars), C_12_BZK (light grey bars) and C_12_PB (dark grey bars). LD_50_ concentrations of each surfactant are normalized with respect to the CMC. Data are presented as mean ± SD of at least 3 independent experiments, each of which was done in triplicate.

The results in Figure 4 show that for the homologous series of cationic surfactants examined, the toxicity to mammalian cells was not linearly dependent upon the surfactant hydrophobic chain length. The toxicity ranking of the surfactants studied towards MDCK, Caco-2 and HeLa cells, normalized with respect to their respective CMCs, was C_10_TAB>C_12_TAB>C_16_TAB>C_14_TAB (see, e.g., Figure 4). In the case of FSDC dendritic cells the ranking was similar for 20 and 60 minutes but for longer exposures times C_14_TAB was slightly more toxic than C_16_TAB. The effect of the polar head group of the cationic surfactants was also evaluated by comparing the effects of three surfactants with a 12 carbon *n*-alkyl chain: C_12_TAB, C_12_BZK and C_12_PB. C_12_BZK and C_12_PB are between 2 to 5 times more toxic (using the CMC-normalized concentration scale) than C_12_TAB in all cell lines tested (Figure 5). The reason for this difference is unclear but could be related to the larger polar head groups (C_12_BZK and C_12_PB) or to the more delocalized positive charge (C_12_PB) and may merit further investigation.

All the cationic surfactants tested had bromide as a counter-ion. Since the bromide ion itself is known to be cytotoxic and the chloride ion is less so, the toxicity of C_12_PB and C_12_-pyridium chloride to MDCK cells was evaluated *in vitro*. No significant difference was found between the two of them (data not shown). It is probable that at the concentrations used in this work the anion toxicity is not a relevant parameter.

### Comparison of the MTT and the LDH leakage assays

**Figure 6:**
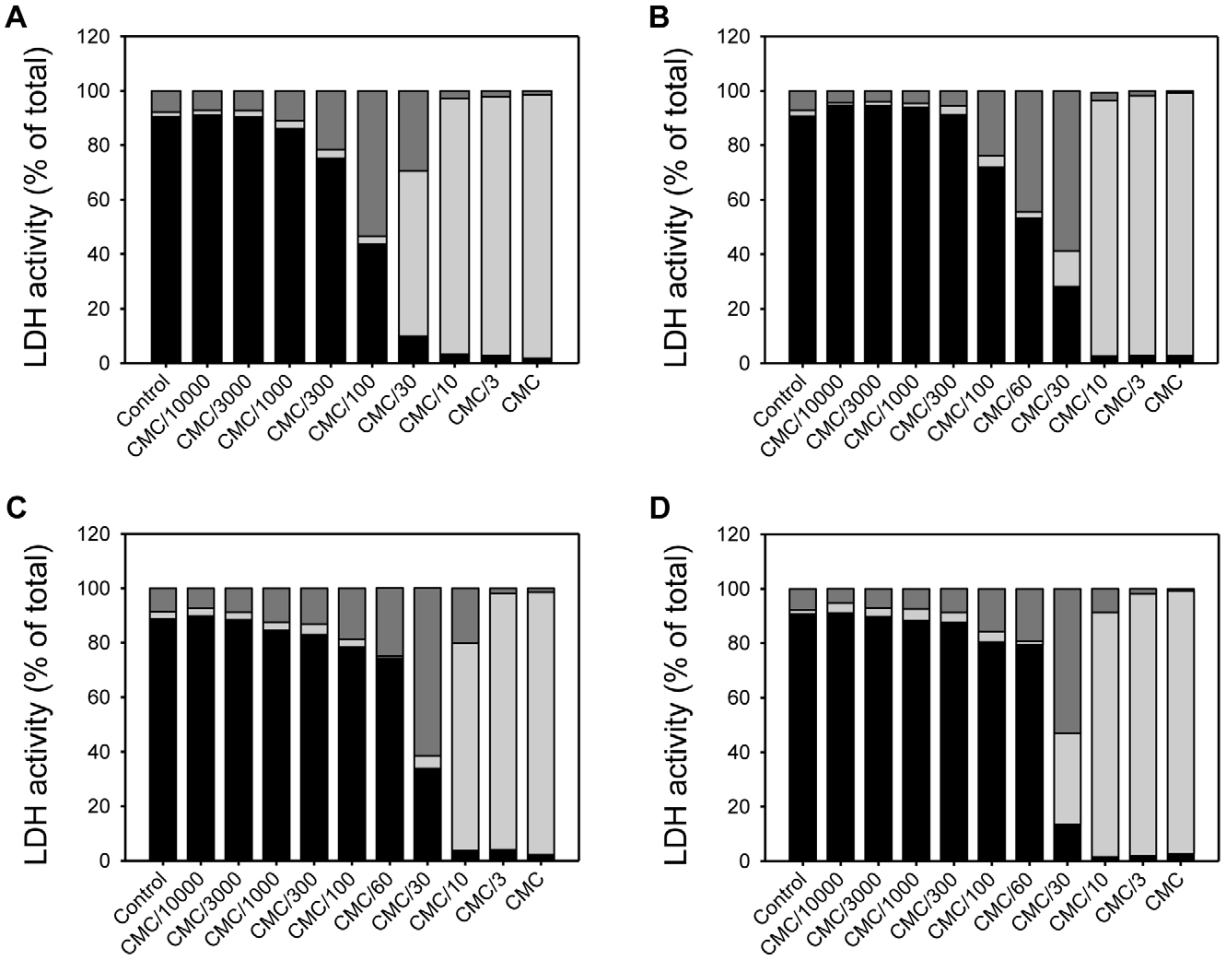
C_n_TAB surfactants toxicity towards HeLa cells as evaluated by the LDH Leakage Assay. HeLa cells were exposed to different concentrations of C_10_TAB (A), C_12_TAB (B), C_14_TAB (C) and C_16_TAB (D) for 540 minutes. After that, the incubation media was collected and replaced by fresh media and the cells were kept for more 24 hours. At the end of the experiment the culture media was collected and the cells were lysed. The LDH activity of the incubation media (light grey bars), cell culture media (dark grey bars) and cell lysates (black bars) was determined and expressed as percentage of the total activity for each condition. Concentrations are normalized with respect to the CMC of each surfactant. Data are presented as mean of at least 3 independent experiments, each of which was done in triplicate.

In order to confirm the toxicity results obtained by the MTT assay, we have measured the activity of the cytoplasmic enzyme lactate dehydrogenase (LDH) in the extracellular medium, which evaluates plasma membrane integrity. HeLa cells were exposed for 540 minutes to C_n_TAB surfactants, as describe for the MTT assay. After that, the incubation medium was collected and replaced by fresh surfactant-free medium and the cells were kept in culture a further 24 hours. At the end of the experiment the culture medium was collected and the cells were lysed. The LDH activity of the incubation medium, cell culture medium and cell lysates was determined and expressed as percentage of the total activity for each condition. The cell viability results obtained with the MTT assay (Figure 2) correlated well with the observed release of LDH from the cells (Figure 6). However, in the case of C_10_TAB and C_12_TAB, the LD_50_ values obtained with the LDH leakage assay were significantly higher than with the MTT assay (Figure S1).

From the LDH results we can also observe that surfactant concentrations close to the critical micelle concentration cause acute toxicity while lower concentrations can lead to a persistent post-exposure toxicity. Since in the majority of published papers concerning cell toxicity the tests were performed immediately after surfactant treatment, the toxic concentrations in those reports should probably be considered to be underestimated.

### Spermicidal activity of surfactants

**Figure 7:**
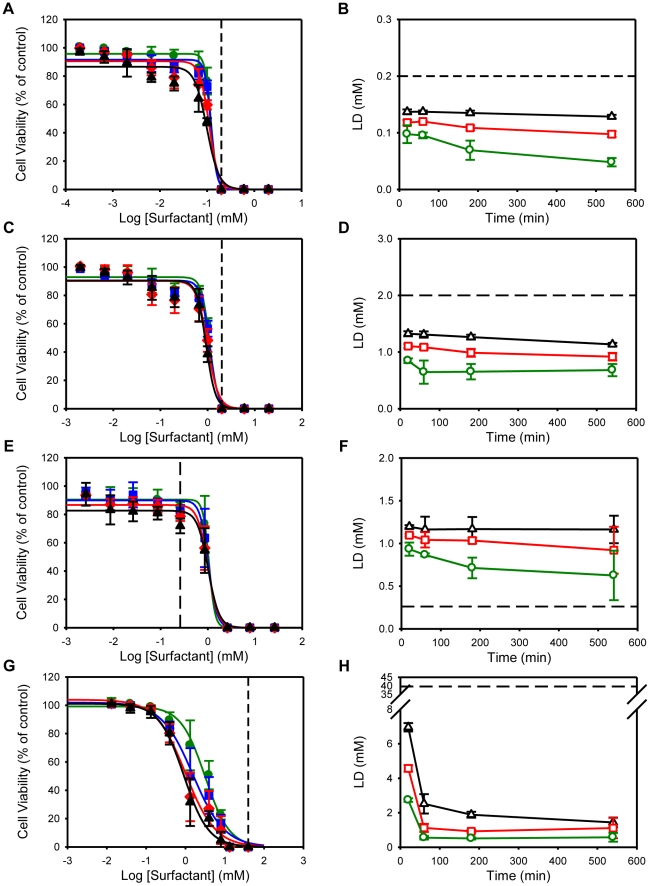
Effect of different surfactant classes on human sperm cells viability. Cell viability was assessed by the LIVE/DEAD® Sperm Viability Kit, as described in the Materials and Methods, immediately after the cells had been exposed to different concentrations of surfactants for 20, 60, 180 and 540 minutes. Other details are as in Figure 1.

Currently the most used spermicides in Europe and in the United States are surfactants, nonoxynol-9 (a nonionic surfactant similar in structure to TX-100) being the most common. In Europe spermicidal gels also commonly have benzalkonium chloride [39]. We tested spermicidal activity of the surfactants by percentage of sperm heads with only SYBR-14 (green fluorescence – live sperm) or also with propidium iodide (red fluorescence – dead sperm), immediately after surfactant exposure. The sperm cells were incubated for 20, 60, 180 and 540 minutes with different surfactants. The results shown in Figure 7 were similar to the ones obtained for the non-polarized HeLa cells, with the exception of C_16_TAB that was not toxic at concentrations below its CMC (data not shown). The efficacy of the spermicidal activity depends on the balance between the concentration required for spermicidal function and the concentration that causes damage to the vaginal epithelium, and this can be measured by a therapeutic index (defined as the ratio of the LD_50_ value for Caco-2 cells to the LD_50_ value for sperm cells, both treated with the same surfactant for the same exposure time) [40], [41]. When dealing with drugs, for example, the desired therapeutic index (as defined above) is usually ≥10. As seen in Table 3, the surfactants studied presented rather narrow therapeutic indices, meaning that, in principle, their eventual use as spermicides for topical intravaginal use should, at the very least, be carefully controlled.

**Table 3:** Therapeutic Index of Surfactants with respect to spermicidal activity.

Surfactant		Therapeutic Index after Exposure Time of			
		20 min	60 min	180 min	540 min
Non-ionic	TX-100	1.4±0.27	0.8±0.05	0.7±0.05	0.7±0.05
Zwiterionic	DDPS	2.0±0.06	2.0±0.08	1.5±0.24	1.2±0.10
Anionic	SDS	0.7±0.08	0.4±0.01	0.1±0.01	0.1±0.04
Cationic	C_10_TAB	3.1±0.22	6.2±1.35	4.2±0.51	1.8±0.94
	C_12_TAB	3.2±0.13	2.7±0.18	2.9±0.85	1.5±0.36
	C_14_TAB	1.8±0.07	2.7±1.14	3.6±1.58	2.6±0.65
	C_16_TAB	0.3±0.04	0.2±0.01	0.1±0.02	0.1±0.01
	C_12_PB	6.8±0.64	5.4±0.47	2.4±0.32	2.7±1.15
	C_12_BZK	5.8±1.86	4.0±0.94	5.7±5.44	4.4±1.42

The therapeutic index (TI) was defined as the LD_50 (Caco-2_)/LD_50 (Sperm)_. Data are shown as mean ± SD of at least 3 independent experiments.

## Discussion

The first criterion to be considered in the evaluation of the safety of topical vaginal microbicides is their toxicity to the cervicovaginal mucosa. On ethical grounds any such evaluation should begin with an *in vitro* screening before using animal models and human studies.

In this work we have screened the *in vitro* effects of concentration, exposure-time and surfactant structure on the viability of mammalian cells with characteristics mimicking the different cell types that exist in the human cervicovaginal mucosa. Earlier studies identified vaginal columnar epithelial cells as the most important site of damage caused by the surfactants Nonoxynol-9 [10], [20] and C31G [19] in humans. Our studies were thus performed on two laboratory models of mammalian columnar epithelial cells, namely, fully polarized MDCK and Caco-2 cells grown to confluence. These cell cultures have well-formed tight junctions and are commonly used as models of “tight epithelia” in laboratory trans-epithelial drug transport studies. Non-polarized cells, in particular dendritic cells, have previously been identified as the primary sites of viral infection in the vagina [3], [34], [35], [36]. We, therefore, also studied surfactant toxicity to dendritic cells under non-confluent conditions. As a model for confluent but non-polarized cells, vaginal “epithelial-like” HeLa cells were also included in this study. A comparison of these two (fully-polarized and non-polarized) cell types is in itself instructive since the exposed cell surfaces are expected to be significantly different in membrane order [42] and, therefore, with regard to surfactant partition into [30], [31] and translocation across them [32]. The results reported here indicate that all surfactants used in this study revealed concentration and time-dependent toxic effects but exhibited different degrees of toxicity depending on the chemical nature of their polar head, which is in agreement with previous *in vitro* [17], [43] and *in vivo* studies [44]. For Triton X-100, DDPS and SDS, cytotoxicity was not observed up to concentrations close to the Critical Micelle Concentration (CMC), in both non-polarized (epithelial-like HeLa and dendritic FSDC) and polarized epithelial (MDCK and Caco-2) cell lines, whereas the toxicity of cationic surfactants occurred at concentrations very much lower than the surfactant CMC, non-polarized cells being around 10-times more sensitive to these surfactants than polarized epithelial cells. We interpret this result to mean that TX-100, DDPS and SDS act mainly at the level of the plasma membrane of the cells probably by causing structural changes at the level of the membrane or even its dissolution, as expected at concentrations close to the surfactant CMC [45]. On the other hand, the cationic surfactants are probably toxic at a more subtle level, toxicity being at concentrations that are not sufficient to cause significant damage to the physical integrity of the membranes. These effects could even be at the intracellular level, conditioned by membrane partitioning [30], [31] and/or translocation [32] across the membranes. The highly ordered apical membranes of fully polarized cells are the only membrane exposed to the surfactants in confluent polarized cell cultures and intact epithelia whereas non-polarized cells have significant amounts of less-ordered membrane domains exposed.

For the homologous series of cationic surfactants examined, the results show that the toxicity to mammalian cells was not linearly dependent upon the surfactant hydrophobic chain length. This observation may have complex reasons related to different affinities of the surfactant for the different, possibly multiple, sites of their action. Without precise information concerning these affinities, something that we are working to obtain, any further discussion of this aspect would be speculative. The effect of the polar head group of the cationic surfactants was also evaluated; C_12_BZK and C_12_PB, which have the larger polar head groups and more delocalized charge, were between 2 to 5 times more toxic than C_12_TAB in all cell lines tested. Delocalized charge on the surfactant head group makes its ionic radius considerably larger and reduces the work required for translocation of the polar group from one side of the membrane to the other [46], [47].

Though there have been a very large number of reports concerning the disinfectant properties of surfactants, their mechanism of action is still not fully understood. Attempts to use surfactants in STI prophylaxis relied upon their capacity to destroy viral and bacterial membranes but did not seem to take into account, what in hindsight appears all too obvious, that if they destroyed those membranes they would also destroy the membranes of cells of the vaginal epithelium. However, destruction of cell membranes is not the only mechanism of surfactant toxicity as is evidenced in the case of cationic surfactants. As argued above, their toxic effects probably do not involve gross disassembly of the cell membrane but rather some more subtle effects. Candidate mechanisms that have been proposed in the literature include modulation of membrane curvature elastic stress and consequent reduction of membrane-bound protein activity [48], alteration of the electrostatic surface potential of membranes [38], or interaction with anionic polymers (DNA and RNA) in the cytoplasm or cell nucleus following translocation across the cell plasma membrane [49]. Cationic surfactants are known to bind strongly to DNA and RNA [50], [51] and induce drastic conformational changes in the structure of these polymers [52].

The cell viability results obtained with the MTT assay correlated well with the observed release of LDH from the cells. However, in the case of C_10_TAB and C_12_TAB, the LD_50_ values obtained with the LDH leakage assay were slightly but significantly higher than with the MTT assay. This can be explained by the nature of each assay: the LDH leakage assay, which evaluates the loss of intracellular LDH and its release into the culture medium, is an indicator of irreversible cell death either due to cell membrane damage directly caused by the surfactants or due to loss of plasma membrane integrity posterior to cell death due to reasons that have nothing to do with direct membrane damage by the surfactants. On the other hand, the MTT assay evaluates the metabolic capacity of the cell in reducing MTT to formazan. Our results suggest that the molecular targets of the C_n_TAB surfactants may be different depending on the length of their hydrophobic chain. Amphiphiles with single short hydrocarbon chains insert faster into and translocate faster across lipid bilayer membranes than amphiphiles with longer hydrocarbon chains (R. Cardoso et al. (2011), submitted for publication). Also, for amphiphiles with the same polar head groups, the longer the hydrocarbon chain the higher the partition coefficient between the membrane and the aqueous phase. Thus, the cationic surfactants with shorter hydrophobic chains are expected, at equilibrium, to be at a higher concentration within the cytoplasm than their analogues with longer hydrocarbon chains while the latter are expected to be more concentrated in the cell membranes. From a kinetic perspective the shorter hydrocarbon chain surfactants would also be expected to reach sites of metabolic activity such as polynucleotides and mitochondrial membranes faster than their longer analogues.

Our results also show that surfactant concentrations close to the critical micelle concentration cause acute toxicity while lower concentrations can lead to a persistent post-exposure toxicity. It has been proposed that high concentrations of surfactants cause necrosis whereas low concentrations induce apoptosis [53]. Enomoto and co-workers [54] demonstrated that some cationic surfactants, such as benzalkonium chloride, induce apoptosis at very low concentration (below CMC) by causing structural changes on the plasma membrane, such as phosphatidylserine translocation, but anionic and amphoteric surfactants could not induce apoptotic cell death.

There is an urgent need for cheap, safe, easy-to-use and easy-to-store woman-controlled topical vaginal microbicides that could be used by people in poor regions all over the world. Surfactants, if safe and effective, would be ideal candidates since they satisfy most of the other stipulated criteria. If microbicide gels could also prevent unintended pregnancies, the interest of women in using them might be substantially higher. For that reason we have tested the spermicide effect of surfactants. Most of the surfactants tested in this work are clearly of no use as spermicidal contraceptive agents since they are as toxic to sperm as they are to other cells typically found in the vagina. Only C_12_PB and C_12_BZK show slightly more toxicity to sperm than they do to polarized epithelial cells but even in these two cases their narrow therapeutic indices imply that their use as contraceptive agents should be implemented with great care. This result was somehow unexpected for C_12_BZK which is commonly used, and at surprisingly high concentrations, as a contraceptive in contraceptive sponges [55] and condoms [39]. Our results show that the *in vitro* LD_50_ for the surfactants examined here are some orders of magnitude lower than the “contraceptive” doses used in these devices. Our present results, taken in combination with the results of earlier *in vivo* studies from other laboratories [44], [56], raise serious doubts as to whether many if not most of the contraceptive sponges and spermicide gels on the market today should be licensed for use at all. Clearly, when surfactants are used as contraceptive agents or microbicides for human use, the LD_50_ values reported here and in previous *in vivo* works [44], [56] should serve as some sort of guideline.

In conclusion, the systematic study of structure-toxicity relationship in laboratory cell culture models is of the utmost importance in the understanding of the mechanisms underlying surfactant use in prophylaxis against STIs and as contraceptive agents. The detailed approach we have used in this work should be a mandatory first-line screening of possible microbicide candidates in research concerning STI prophylaxis. Altogether, our results contribute to the understanding of the mechanisms involved in surfactant toxicity and can be used to make predictions about the safety of these molecules, which would be helpful in the design of new, more effective and less harmful surfactants that could be used as broad-spectrum microbicides in vaginal gels. Our results clearly show that among the surfactants tested only cationics show selectivity with respect to mammalian cell types – they are significantly more toxic to non-polarized than they are to polarized cells. Similar selectivity was previously reported by us [17] in a comparison of bacterial and yeast cells with polarized mammalian epithelial (MDCK) cells. This feature should be considered in future surfactant design. We are presently working on this aspect and have preliminary results that confirm this hypothesis. Finally, it should be considered that a single “magic bullet” solution may never be found for prevention of STIs and that synergistic approaches, targeting simultaneously pathogens and host cells, must be sought. Specially designed surfactants could be important players in the desired synergy.

## Materials and Methods

### Reagents

All cell culture reagents were purchased from Invitrogen Corporation (Carlsbad, CA, USA). The LIVE/DEAD® Sperm Viability Kit (L-7011) was purchased from Molecular Probes (Eugene, OR, USA) and the Cytotoxicity Detection Kit^PLUS^ (LDH assay) was obtained from Roche Applied Science (Germany). All chemicals were of the highest commercially available purity and were used as received. The 3-(4,5-Dimethylthiazo-2-yl)-2,5-diphenyltetrazolium bromide (MTT), the nonionic surfactants Triton X-100 (TX-100) and rac-1-lauroylglycerol (Monolaurin), the anionic sodium dodecyl sulfate (SDS), the zwitterionic N-dodecyl-N,N-dimethylammonium-propanesulfonate (DDPS) and the cationic surfactants dodecyl-trimethylammonium bromide (C_12_TAB), tetradecyl-trimethylammonium bromide (C_14_TAB), hexadecyl-trimethylammonium bromide (C_16_TAB), N-dodecylpyridinium bromide (C_12_PB), N-dodecylpyridium chloride and benzyldodecyldimethylammonium bromide (C_12_BZK) were obtained from Sigma-Aldrich (St. Louis, MO, USA). Decyltrimethylammonium bromide (C_10_TAB) was from Fluka. All others chemicals were from Sigma-Aldrich (St. Louis, MO, USA).

### Biological Materials

Caco-2, a colorectal adenocarcinoma human cell line, and HeLa, a human cervical adenocarcinoma cell line, were purchased from ATCC. The MDCK II immortalized canine columnar epithelial cell line was a gift of Professor Kai Simons (Max-Planck-Institute of Molecular Cell Biology and Genetics, Dresden, Germany) and the fetal mouse skin-derived dendritic cell line was kindly supplied by Dr. G. Girolomoni (Laboratory of Immunology, Instituto Dermopatico dell'Imacolata, IRCCS, Rome, Italy). Patients were recruited from the Fertility Clinic (University Hospitals of Coimbra, Portugal) and were undergoing routine semen analysis or fertility treatment. Written informed consent was obtained from all patients, who signed informed consent forms for this purpose, approved by the Institutional Review Board (IRB) of the University Hospitals of Coimbra. All human material was used in accordance with the appropriate ethical guidelines provided by the IRB of the University Hospitals of Coimbra, who also approved the study. Fresh semen samples were obtained by masturbation after 3–5 days of sexual abstinence from healthy males undergoing routine semen analysis or fertility treatment and seminal analysis was carried out in conformity with the WHO recommendations [57]. Semen samples were prepared by density gradient centrifugation using Isolate Sperm Separation Medium (Irvine Scientific, Santa Ana, CA, USA) and only samples with normal WHO parameters were used in the study.

### Determination of the Critical Micelle Concentration (CMC)

The CMC was measured as described elsewhere [58]. The solutions of surfactants were prepared in a saline buffer with similar pH and saline composition as the OptiMEM media that was used in cell incubation. The buffer composition was 1.8 mM CaCl_2_, 0.8 mM MgSO_4_, 5.3 mM KCl, 26.2 mM NaHCO_3_, 117.2 mM NaCl, and 1.0 mM NaH_2_PO_4_.H_2_O, pH 7.3. Measurement of the CMC of C_12_PB used a similar protocol but the fluorescent probe N-phenyl-I-naphthylamine was replaced by boron-dipyrromethene (Bodipy) at a final concentration of 0.9 µM (in 99% ethanol). In this case excitation was at 483 nm and emission intensity was recorded at 510 nm. As a control, the CMC of C_12_BZK was measured with both Bodipy and NPN with essentially identical results.

### Cell Culture and Experimental Treatment

MDCK cells were grown on 96 MW plates (Corning Costar, Corning, NY) for 3 days in MEM with 10% FCS and 100 units/ml of penicillin, 100 µg/ml streptomycin. Caco-2 cells were plated in 96 MW plates and kept for 10 days in D-MEM/F-12 with GlutaMAX^TM^, supplemented with 10% FCS, 100 units/ml of penicillin, 100 µg/ml streptomycin, 1% sodium pyruvate and 1% of non-essential amino acids. After that time, both cell lines were confluent and polarized. HeLa cells were seeded in 96 MW plates and grown in D-MEM/F-12 with GlutaMAX™ with 10% FCS and 100 units/ml of penicillin, 100 µg/ml streptomycin for 24 hours, when they reached confluence. The dendritic FSDC cells were grown in 96 MW plates for 24 hours, until they were 80% confluent, in IMDM with 10% FCS and 100 units/ml of penicillin, 100 µg/ml streptomycin [59], [60]. The cells were then exposed to different concentrations of the surfactants for 20, 60, 180 and 540 minutes. After that, the incubation media was collected and replaced by fresh media and the cells where kept for more 24 hours. Stock solutions of surfactants were prepared in OptiMEM cell culture media, without serum and antibiotics, as multiples of their CMC.

### Evaluation of in vitro toxicity of surfactants towards mammalian cells

Cell Viability was assessed 24h after the cells had been exposed to different concentrations of surfactants for 20, 60, 180 and 540 minutes. Viable cells were measured by their ability to reduce the tetrazolium salt, 3-(4,5-dimethylthiazole-2-y)-2,5-diphenyltetrasodiumbromide (MTT), to a formazan dye detectable by spectrophotometric analysis in a microplate reader.

The activity of the cytoplasmic enzyme lactate dehydrogenase (LDH) in the extracellular medium, which evaluates plasma membrane integrity, was determined in HeLa cells. After the treatment with C_10-16_TAB for 540 minutes, the incubation medium was collected and replaced by fresh medium and the cells where kept for more 24 hours. At the end of the experiment the culture medium was collected and the cells were lysed. The LDH activity of the incubation media, cell culture media and cell lysates (intracellular content) was determined with a commercial kit from Roche Applied Science (Detection Kit^PLUS^ (LDH)), according to the standard protocol provide by the supplier.

### Evaluation of surfactant activity on sperm membrane integrity/viability

The percentage of viable cells was detected using the viability kit, consisting of two DNA-binding fluorescent dyes: SYBR-14 (which is membrane-permeant and thus stains all sperm with bright green fluorescence in the nucleus) and propidium iodide (PI; that only penetrates sperm nuclei with compromised membrane integrity, fluorescing red and overwhelming the SYBR-14 signal) [61]. Primary stock solutions were prepared in water (PI) or in DMSO (SYBR-14), aliquoted, stored at −20°C and protected from light. Secondary stock solutions were prepared in phosphate-buffered saline (PBS; pH 7.2), immediately prior to use. Live sperm suspensions (10x10^6^ sperm/mL) were incubated in a PBS-Glucose-BSA (PBS-Glu-BSA) medium (an appropriate sperm culture medium constituted of 1 g/L of glucose and 3 g/L of BSA) with SYBR-14 (100 nM) and PI (240 nM) for 10 minutes, at 37°C, in the dark. Samples were then mounted on a microscope slide and immediately observed under a Zeiss Axiophot II microscope (Carl Zeiss, Göttingen, Germany) and 200 sperm were counted and analysed.

### Statistical analysis

Results were expressed as the means ± standard deviations (SD) as indicated in the Figure and Table legends. The cell viability dose-response curves were fitted to a four parameter sigmoid equation [24], [25] through computer-assisted curve fitting (SigmaPlot® 11.0, SPSS Science Inc., Chicago, IL). The fitted equation was: 
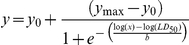
, where *x* is the surfactant concentration, *y*_max_ is the maximal percentage of cell viability, *y*_0_ is the basal cell viability, *b* is the curve slope and *LD*_50_ is the lethal dose 50 (LD_50_). From these data, it was possible to calculate the lethal dose (LD) 90, 50 and 10 concentrations for the each individual data set. The LD concentrations were plotted against time of exposure to the surfactants and fitted to a mono-exponential decay equation using SigmaPlot® 11.0. The fitted equation was: 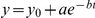, where *t* is time, *y*_0_ is the LD concentration at an exposure time of 0 minutes, 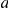 is the difference between *y*_0_ and the asymptotic LD concentration at “infinite” exposure time and *b* is the decay constant.

## Supporting Information

Figure S1 **Comparison of LDH leakage assay and MTT assay in HeLa cells.** The cells were exposed to C_10_TAB (A), C_12_TAB (B), C_14_TAB (C) and C_16_TAB (D) for 540 minutes. Cell Viability was assessed by the MTT assay (grey bars) or LDH assay (black bars) 24h after the cells had been exposed to different concentrations of surfactants and cell viability is expressed as percentage of the viability of control cells or as percentage of the LDH intracellular activity of control, respectively. The data of each independent experiment was fitted to a four parameter logistic equation and the LD_10_, LD_50_ and LD_90_ concentrations were determined for each time point (E). Data are presented as mean ± SD of at least 3 independent experiments, each one done in triplicate. Two-way ANOVA (Bonferroni's post-test): **P<0.01 and *P<0.05, significantly different from LDH results; ns, not significant.
(TIF)
