# Supplementary figures and images for: In Vitro Surfactant Structure-Toxicity Relationships: Implications for Surfactant Use in Sexually Transmitted Infection Prophylaxis and Contraception
Source: PLoS One. 2011 May 16;6(5):e19850. doi: 10.1371/journal.pone.0019850 (PMC3095630; doi:10.1371/journal.pone.0019850)

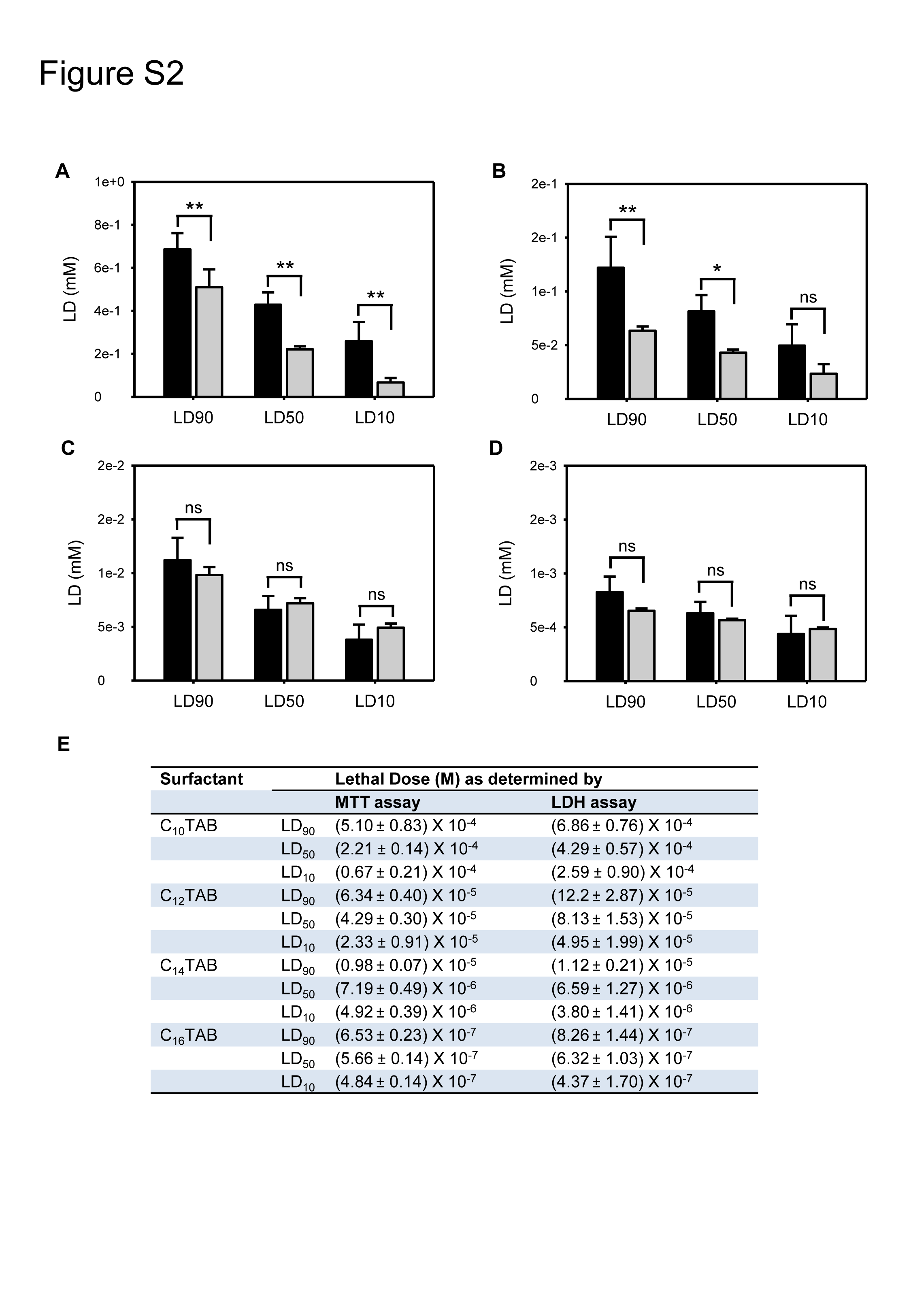

Supplement: Figure S1 — Comparison of LDH leakage assay and MTT assay in HeLa cells. The cells were exposed to C10TAB (A), C12TAB (B), C14TAB (C) and C16TAB (D) for 540 minutes. Cell Viability was assessed by the MTT assay (grey bars) or LDH assay (black bars) 24h after the cells had been exposed to different concentrations of surfactants and cell viability is expressed as percentage of the viability of control cells or as percentage of the LDH intracellular activity of control, respectively. The data of each independent experiment was fitted to a four parameter logistic equation and the LD10, LD50 and LD90 concentrations were determined for each time point (E). Data are presented as mean ± SD of at least 3 independent experiments, each one done in triplicate. Two-way ANOVA (Bonferroni's post-test): **P<0.01 and *P<0.05, significantly different from LDH results; ns, not significant. (TIF) [file pone.0019850.s001.tif]
